# Preparedness of primary health care facilities on implementation of essential non-communicable disease interventions in osun state south-west Nigeria: a rural–urban comparative study

**DOI:** 10.1186/s12913-023-09138-8

**Published:** 2023-02-14

**Authors:** Adebowale Femi Akinwumi, Olapeju Adefunke Esimai, Olujide Arije, Temitope Olumuyiwa Ojo, Oluwaseun Taiwo Esan

**Affiliations:** 1 Department of Community Medicine, Faculty of Clincal Sciences, Ekiti State University, Ado Ekiti, Ekiti State Nigeria; 2 Department of Community Health, Obafemi Awolowo University, Ile Ife, Osun State Nigeria

**Keywords:** Preparedness, Non-communicable diseases, Primary Health Care, Health system, Basic equipment, Diagnostics, Essential medicines, Nigeria

## Abstract

**Background:**

Global response to the growing burden of non-communicable diseases (NCDs) in developing countries includes the development of WHO Package of Essential Non-communicable Disease Interventions (WHO PEN) for Primary Health Care (PHC). The study assessed the level of preparedness of PHC facilities on implementation of essential NCD interventions in rural and urban Local Government Areas (LGAs) of Osun State, Nigeria.

**Methods:**

The study was a comparative cross-sectional survey. Information was collected from heads of 33 rural and 33 urban PHC facilities and through direct observation on the domains of staff training, basic equipment, diagnostics and essential medicines for cardiovascular diseases, diabetes and chronic respiratory diseases (CRDs) using a semi-structured interviewer administered questionnaire.

**Results:**

Manual sphygmomanometer was found in similar proportions (84.8%) of PHC facilities in rural and urban LGAs. Glucometer was available in 45.5% of the PHC facilities in urban and 33.3% of the PHC facilities in the rural LGAs, the difference was not statistically significant (χ2 = 1.015; *p* = 0.314). Basic equipment for CRDs were not available in majority of PHC facilities in both locations. Moduretic tablets were the most reported essential NCD medicines, available in 15% of PHC facilities in rural LGAs and none in urban LGAs. The anti-diabetic medicines were not available in any of the PHC facilities in both locations. More than 90% (≥ 30) of the PHC facilities in both locations were not prepared to implement essential interventions for each NCD across domains of staff training and essential medicines. Overall, 97.0% of the PHC facilities in the rural LGAs and all the PHC facilities in urban LGAs were not prepared on implementation of essential interventions for the three NCDs.

**Conclusion:**

The level of preparedness of the PHC facilities on implementation of essential NCD interventions in the rural and urban LGAs of Osun State is very low. Government needs to strengthen the PHC system by providing needed essential medicines, basic diagnostics, equipment, and training of clinical health care workers for implementation of essential NCD interventions in the state.

**Supplementary Information:**

The online version contains supplementary material available at 10.1186/s12913-023-09138-8.

## Introduction

Non-communicable diseases (NCDs) are now leading causes of mortality and morbidity. By 2030, NCDs are projected to become the most common causes of death in Africa [1, 2]. According to the World Health Organization (WHO), cardiovascular diseases, diabetes, cancers, and chronic respiratory disease are the major NCDs [3, 4]. These four NCDs also share four behavioural risk factors, namely tobacco use, unhealthy diet, physical inactivity and harmful use of alcohol [4]. The 2018 WHO report on non-communicable diseases showed that NCDs caused 75% of all premature adult deaths among the age group (30–69 years) [5]. The burden of these diseases falls mainly on low and middle income countries (LMICs) where over three quarters of all NCD deaths occur, amounting to 31.4 million deaths annually [4].

The burden of NCDs in Nigeria is on a steady increase [6–12]. Oladapo et al reported that among adult population in South-West Nigeria, 20.8% were hypertensive with BP ≥ 140/90 mmHg, 42.3% of the men and 36.8% of the women had BP ≥ 130/85 mmHg; 2.5% had diabetes, 3.9% had general obesity, 14.7% had abdominal obesity, 3.2% were physically inactive, and 1.7% smoked cigarettes. Overall, 12.9% of the subjects were found to have at least one CVD risk factor. [12] Also, as at 2017, 29% of all deaths in Nigeria were attributable to NCDs [5].

There is limited access to quality NCD care and services among people of low socioeconomic class who are suffering from NCDs primarily due to wide inequalities in access to high quality health care services compared to those of high socioeconomic class in LMICs [13]. In Nigeria, the diagnosis and management of NCDs occur mostly at the secondary and tertiary levels of health care. The secondary level of health care in the country is poorly funded, ill-staffed and poorly equipped by the state governments while the tertiary care majorly, under the federal government, is financially and physically inaccessible to majority of the population. In terms of cost-effectiveness of essential NCD services and interventions, it has been noted that NCD services delivered at the tertiary level are generally not cost-effective [14].

One of the objectives of the WHO Global Action Plan for the prevention and control of NCDs 2013–2020 is to integrate very cost-effective NCD interventions into the basic Primary Health Care package with referral systems to all levels of care to advance the universal health coverage agenda [15]. This highlights the critical role PHC is expected to play in the control and prevention of NCDs and modification of behavioural risk factors especially among LMICs. One of the nine voluntary targets of this Global Action Plan is that each country should have 80% availability of the affordable basic technologies and essential medicines, including generics, required to treat major NCDs in both public and private facilities [15].

The WHO Package of Essential Non-communicable Disease Interventions (WHO PEN) for Primary Health Care in low-resource settings was developed in the year 2010 [16]. It was an innovative and action-oriented response to the challenges posed by the rising burden of NCDs. It is a prioritized set of cost-effective interventions that deliver acceptable quality of care, even in resource-poor settings. One objective of WHO PEN is to reinforce health systems by contributing to the building blocks of the health system [16]. The Nigerian Federal Ministry of Health adopted WHO PEN in year 2011, but it is yet to be fully integrated into PHC services [17, 18].

The main requirements for preparedness of PHC facilities in both rural and urban settings to implement essential NCD interventions include availability of basic technologies (equipment and diagnostics), essential medicines, trained health personnel, health information system, sustainable health financing systems, and referral systems [16, 19–21].

The WHO strongly advocates that implementing essential NCD interventions under PHC has the potential to prevent NCD complications such as heart attacks, strokes, blindness, amputations, and renal disease, through early detection and treatment of people at high risk [16, 19]. A functional PHC system promotes greater access to essential health services and guarantees better quality of care for majority of the population [22]. The PHC emphasizes greater focus on prevention and health promotion, early diagnosis and management of health problems including NCDs [22]. The PHC system is a proven strategy for achieving cost-effective health care delivery, ensuring equitable access to health services and a veritable tool for achieving universal health coverage.

The preparedness of primary health care facilities in rural and urban settings to respond to the needs of people with NCDs in LMICs, Nigeria inclusive is not well studied [19, 23]. While few studies had determined preparedness [23] or capacity [19] of primary level health facilities to offer essential services for selected NCDs on individual basis and only across each component domain, this present study, aimed to establish an overall level of preparedness across all the domains combined for three major NCDs (cardiovascular diseases, diabetes mellitus and chronic respiratory diseases) identified by the WHO as leading causes of adult mortality and morbidity worldwide. This study assesses and compares the preparedness of PHC facilities, and associated factors, on implementation of essential NCD interventions in rural and urban LGAs of Osun State, Nigeria.

## Materials and methods

### Study setting

The study was carried out in Osun State, South-West Nigeria. The state covers an area of approximately 14,875 sq. kilometers. It is landlocked, bounded by 5 other states, namely Ogun, Kwara, Oyo, Ondo and Ekiti States [24]. It has a projected population of 5.491 million in 2021 [25]. The State has 30 Local Government Areas (LGAs) in three senatorial districts [24]. Based on population density, the LGAs are grouped into 16 rural and 14 urban LGAs [25]. Most of the inhabitants in the state engage in farming and trading [26]. There are two tertiary health care facilities, and 52 secondary health facilities in the state [26]. Also, there are 406 private health facilities in form of medical centers, hospitals, clinics, maternity and convalescent homes [26]. There are 762 PHC facilities fairly evenly distributed across all the senatorial districts and LGAs. The number of health personnel in the PHC facilities in the state include 25 doctors, 224 nurses and midwives, 192 Community Health Officers (CHOs), 918 Senior Community Health Extension Worker (CHEWs), and 355 Junior CHEWs (November, 2017). Also, there are 1,515 voluntary health workers, and 674 traditional birth attendants [27].

At the time of this study, there was no active state-wide NCD programme in the PHC facilities across Osun State. Most efforts to address NCDs in the PHC facilities were individualized (at the discretion of heads of facilities and the Medical Officer of Health (MOH) in the PHC department of each LGA). Although, few years prior to the time of this study, Diabetes Association of Nigeria provided some PHC facilities in selected LGAs with glucometers for screening gestational DM among pregnant women, and few information education and communication (IECs) materials which were sighted during the study.

### Study design

This was a descriptive comparative cross-sectional study of PHC facilities in selected rural and urban LGAs of Osun state. We assessed preparedness of the facilities to implement essential NCD interventions for cardiovascular diseases, diabetes and chronic respiratory diseases (cancer was excluded because of infrequent presentation of cases at the PHC facilities).

### Study population

The study population were heads (or designates) of the PHC facilities. Those included in the study consisted of the heads (or designates) who were nurses, Community Health Officers (CHOs) or Senior Community Health Extension Workers (CHEWs) appointed to oversee the day-to-day activities of the PHC facilities. Those excluded from the study were heads of the PHC facilities that were on leave of absence (≥ 3 months) such as those on terminal leave, maternity leave and study leave during the study.

### Sample size estimation

The minimum sample size was calculated using sample size formula for comparison of two independent proportions [28]. The sample size calculations used 80% power to detect a true difference; assumed a type I error of 5%; and adjusted for a combined non-response rate of 10%, using 75.0% and 37.5% as the proportion of primary health care facilities, in urban and rural areas respectively, that could manage hypertension and diabetes mellitus [23]. Based on this, a total of 66 (33 per group) primary health care facilities were assessed.

### Sampling techniques

A two-stage sampling process was adopted. In the first stage, two rural and two urban LGAs were selected from each senatorial district (Osun West, Osun Central and Osun East) using simple random sampling (balloting), giving a total of six rural and six urban LGAs. In the second stage, five or six PHC facilities were selected from each of the selected rural and urban LGAs using simple random sampling (balloting) to make 33 rural and 33 urban PHC facilities in total. In the selected PHC facility, the head (or designate) was the respondent who provided information on the preparedness of the PHC facility.

### Data collection tools

This instrument was adapted from a standardized WHO Service Availability and Readiness Assessment (SARA) questionnaire, a comprehensive approach to systematically assess and monitor health services [29] and facility capacity assessment questionnaires in WHO Package of Essential Non-communicable Disease Interventions (WHO PEN) for primary health care in low-resource settings earlier used and adapted by Mendis et al., 2012, [16, 19] It had sections on the facility characteristics, the training of health care workers, availability of guidelines on common NCDs, basic technologies (equipment and diagnostics) for diagnosis and management of common uncomplicated NCDs, essential medicines for managing common NCDs, among others.

### Pre-testing of research instrument

The study tool was pretested in six (6) PHC facilities in Ife East LGA which were not selected for the study. This helped to address areas of ambiguity in the questionnaires and determine appropriateness of each question in eliciting the required responses. Questions that were unclear were rephrased and irrelevant ones were removed in line with the study objectives.

### Data collection procedure

Four research assistants who received training on research ethics and data collection assisted with data collection. Data was collected between February and April 2018. Some sections of the health facility questionnaire were answered by the heads (or designees) of PHC facilities while the rest were completed through direct observation (guidelines, IEC materials, functionality of basic equipment, essential medicines and laboratory supplies) by the trained interviewer research assistants.

### Outcomes variables and scoring

The key outcome variable of this study was the level of preparedness of PHC facilities which was determined by the PHC facility service availability and NCD services readiness (the ability of health facilities to offer a specific service measured through consideration of tracer items that include availability of guidelines, IEC materials, trained staff, basic equipment, diagnostics and essential medicines). See Additional file 1: Appendix I.

The availability of health care services (prevention and management) for selected NCDs was expressed as percentage of the overall health facilities where they were available. The availability of an essential item needed for diagnosis and management of selected NCDs at the health facilities was expressed as percentage of the overall health facilities where the tracer essential item was available and/or functional. The functionality of equipment implies that the equipment was available, observed and functional at the PHC facility on the day of visit.

$$Availability\;of\;health\;services\;for\;NCD\;(\%)=\frac{Availability\;of\;health\;services\;for\;NCD\;(\%)}{Total\;No\;of\;PHC\;facility\;surveyed}\ast100\%$$

$$Availability\;of\;essential\;item\;(\%)=\frac{No\;of\;PHC\;facilities\;where\;the\;tracer\;item\;is\;available\;}{Total\;No\;of\;PHC\;facility\;surveyed}\ast100\%$$

PHC facility service readiness is a composite measure. The overall readiness of a facility to provide services for all the selected NCDs was assessed by summing up the services readiness for each of the diseases. Readiness of a facility to provide services for a particular NCD was assessed across the following component domains: staff & training, basic equipment, diagnostics and essential medicines. Service readiness across each domain was assessed based on the availability of the necessary tracer items.

Service readiness or preparedness for prevention and management of a selected NCD across all health facilities was the percentage of facilities providing the health service for the selected NCD using the tracer items on the day of the assessment. This was measured by assigning score of 1 to each available tracer item in a particular component domain [29]. The mean score for each domain was expressed as percentage and computed by summing up the scores for the available tracer items in the domain divided by the number of tracer items per domain multiplied by 100 [29].

$$Mean\;score\;of\;items\;in\;a\;domain\;for\;a\;NCD\;as\;percentage=\frac{\;Total\;score\;of\;items\;in\;a\;domain}{Number\;of\;items\;in\;a\;domain}\ast100\%$$

$$Mean\;score\;of\;items\;in\;all\;domains\;for\;a\;NCD\;as\;percentage\;=\frac{Total\;score\;of\;items\;in\;all\;domains}{Total\;number\;of\;items\;in\;all\;domains}\ast100\%$$

The facilities with percentage readiness score of 50% or more for each NCD were regarded as being prepared for implementation of essential NCD services; and those who have less than 50% were regarded as not prepared to implement essential NCD services.

### Data analysis

Data collected were field-edited so as to ensure all the required items were appropriately answered. Data were entered into spread sheet using Epidata software version 3.1 and analysed using STATA statistical software version 15. Descriptive statistics were carried out and bivariate analysis using Chi-square test (or Fisher exact test where applicable) was used for comparison of categorical variables. The level of significance was determined at *p* < 0.05.

## Results

Table 1 shows the background characteristics of the PHC facilities related to NCD services and availability of NCD services (prevention, diagnosis and/or management) in the rural and urban PHC facilities. The proportion of PHC facilities 26 (78.8%) in the rural LGAs that offered outpatient and maternity inpatients services only was slightly higher than the proportion of PHC facilities 25 (75.6%) in the urban LGAs. Almost nine out of ten PHC facilities in both location areas did not have any specific program for prevention and control of common NCDs. The proportion of PHC facilities 23 (69.7%) in rural LGAs offering hypertension services was higher than the proportion of PHC facilities 20 (60.6%) in the urban LGAs. The difference was not statistically significant (*p* = 0.438). On the contrary, the proportion of PHC facilities 13 (39.4%) in urban LGAs offering diabetes mellitus (DM) services was higher than the proportion of PHC facilities 11 (33.3%) in the rural LGAs. Only a few PHC facilities in both rural and urban LGAs offered chronic respiratory diseases (CRDs) essential services.

**Table 1:** Background characteristics of PHC facilities related to NCD services and availability of NCD services (prevention, diagnosis and/or management)

Characteristics of the PHC facilities	Location (%)		Statistics	
	**Rural** **(** ***n*** ** = 33)**	**Urban** **(** ***n*** ** = 33)**	**χ2**	***P***
**Services offered by the PHC facility**				
Outpatients only	2 (6.1)	2 (6.1)	0.34	0.95
Outpatients and maternity inpatients	26 (78.8)	25 (75.6)		
Outpatient and other inpatient department	2 (6.1)	2 (6.1)		
Outpatient, maternity and other inpatient department	3 (9.1)	4 (12.2)		
**Population served by the PHC facility**				
(Mean pop. and Std. deviation)	10,185 ± 5388	12,729 ± 6657		
**Availability of specific programmes for prevention and control of NCDs in PHC facilities**				
Yes	4 (12.1)	4 (12.1)	0.02	0.96
No	29 (87.9)	29 (87.9)		
**Availability of community activity to support NCD services at PHC facilities**				
Yes	2 (6.1)	1 (3.0)	2.04	0.36
No	26 (95.9)	30 (97.0)		
**Availability of NCD Services**				
** Hypertension**	23 (69.7)	20 (60.6)	0.601	0.438
Yes	10 (30.3)	13 (39.4)		
No				
** Diabetes**				
Yes	11 (33.3)	13 (39.4)	0.262	0.609
No	22 (66.7)	20 (60.6)		
** Chronic respiratory diseases**				
Yes	2 (6.1)	2 (6.1)	0.000^**+**^	1.000
No	31 (93.9)	31 (93.9)		

^+^Fischer’s exact test

*NCD* Non-communicable disease, *PHC* Primary health care

Table 2 shows the comparison of availability of basic equipment for selected NCDs in the PHC facilities in rural and urban LGAs. Stethoscope the most recorded basic equipment, was available in 31 (93.9%) of PHC facilities in the rural LGAs and 32 (97.0%) in PHC facilities in urban LGAs. The manual blood pressure (BP) apparatus was available in PHC facilities in rural and urban LGAs in same proportions (84.8% **/** 84.8%). However, the proportion of PHC facilities 11 (33.3%) in urban LGAs with digital BP apparatus was higher than the proportion of the PHC facilities 5 (12.1%) in the rural LGAs. The difference was not statistically significant (*p* = 0.184). The proportion of PHC facilities 31 (94%) in the urban LGAs that have adult weighing scale was higher than the proportion of PHC facilities 29 (88%) in the rural LGAs. There was no statistically significant difference (*p* = 0.672). There are very few PHC facilities in both rural and urban LGAs with basic equipment (spirometer, peak flow meters, nebulizer and spacer for inhaler) for CRDs.

**Table 2:** Availability of basic equipment needed for diagnosis and management of selected NCDs in PHC facilities in rural and urban LGAs

Basic equipment in PHC facilities	Rural *n* = 33 (%)		Urban *n* = 33 (%)			
	Available	Not available	Available,	Not available	Statistics	
					χ2	*P*
Light source (flashlight acceptable)	13 (39.4)	20 (60.6)	8 (24.2)	25 (75.8)	1.746	0.186
Adult weighing scale	29 (87.9)	4 (12.1)	31 (93.9)	2 (6.06)	0.733^+^	0.672
Measuring tape or height board /stadiometer	24 (72.7)	9 (27.3)	23 (69.7)	10 (30.3)	0.074	0.786
Thermometer	32 (97.0)	1 (3.0)	27 (81.8)	6 (18.2)	3.995^+^	0.105
Stethoscope	31 (93.9)	2 (6.1)	32 (97.0)	1 (3.0)	0.349^+^	1.000
Digital blood pressure apparatus	5 (12.1)	28 (84.9)	11 (33.3)	22 (66.7)	3.387	0.184
Manual blood pressure apparatus	28 (84.8)	5 (15.2)	28 (84.8)	5 (15.2)	0.000	1.000
Peak flow meters	1 (3.0)	32 (97.0)	2 (6.1)	31 (93.9)	0.349^+^	1.000
Spirometers	0 (0.0)	33 (100.0)	2 (6.1)	31 (93.19)	2.063^+^	0.492
Spacers for inhalers	0 (0.0)	33 (100.0)	1 (3.0)	32 (97.0)	1.015^+^	1.000
Nebulizer	0 (0.0)	33 (100.0)	1 (3.0)	32 (97.0)	1.015^+^	1.000

^**+**^Fisher’s exact test

PHC- Primary health care

Table 3 shows the availability of basic diagnostic tests in PHC facilities in the rural and urban LGAs. Diagnostic services including rapid diagnostic testing (RDTs) were available in 32 (97.0%) of PHC facilities in the urban LGAs and 31 (93.9%) of the PHC facilities in the rural LGAs. Glucometer was available in 15 (45.5%) of PHC facilities in the urban LGAs and 11 (33.3%) of the PHC facilities in the rural LGAs. Forty two percent of the PHC facilities in the urban LGAs had more protein dipstick test compared with 11 (33.3%) of PHC facilities in rural LGAs. Three percent of the PHC facilities in both urban and rural LGAs had available test for blood cholesterol. There was no test for serum creatinine in the PHC facilities in rural LGAs unlike 2 (6.0%) of PHC facilities in the urban LGAs had available test for serum creatinine.

**Table 3:** Comparison of availability of basic diagnostics and supplies for diagnosis and management of selected NCDs in the laboratory or service area in the PHC facilities

Basic Diagnostics	Location (%)			Statistics
	**Rural (*n* = 33)**	**Urban (*n* = 33)**	**χ2/ Fisher’s exact test** ***p***	
**Availability of diagnostic services including (RDTs)**				
Yes	31 (93.9)	32 (97.0)	0.349^a^	1.000
No	2 (6.1)	1 (3.0)		
**Availability of Glucometer**				
Yes	11 (33.3)	15 (45.5)	1.015	0.314
No	22 (66.7)	18 (54.5)		
**Availability of urine protein dipstick tests**				
Yes	11 (33.3)	14 (42.4)	0.580	0.447
No	22 (66.7)	19 (57.6)		
**Availability of urine glucose dipstick tests**				
Yes	10 (30.3)	12 (36.4)	0.273	0.602
No	23 (69.7)	21 (63.6)		
**Availability of blood cholesterol tests**				
Yes	1 (3.0)	1 (3.0)	0.000^a^	1.000
No	32 (97.0)	32 (97.0)		

^**a**^Fischer’s exact test

RDTs- Rapid diagnostic tests

**Fig. 1:**
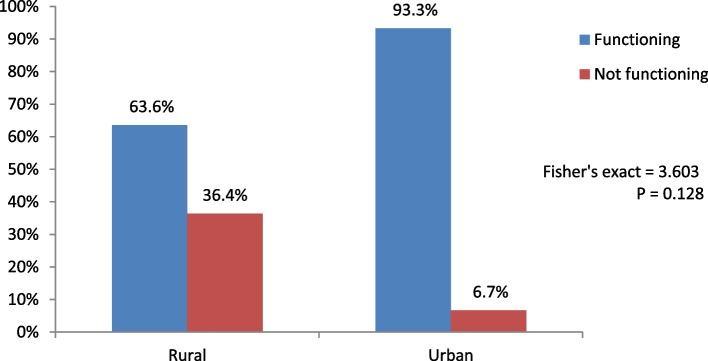
Functionality of available glucometers in PHC facilities in the rural and urban areas

The proportion of glucometer that were functional is displayed in Fig. 1. Among the PHC facilities that have glucometers, the proportion of PHC facilities 14 (93%) in the urban LGAs that have functional glucometers was higher than the proportion of PHC facilities with the functional device 7 (63.6%) in the rural LGAs. This difference was not statistically significant (Fisher's exact = 3.603; p = 0.128).

Table 4 shows comparison of availability of essential medicines for management of selected NCDs in PHC facilities in rural and urban LGAs. There was low availability of essential medicines in the PHC facilities in rural and urban LGAs to implement essential NCD interventions. Essential medicines for DM such as metformin, glibenclamide and insulin were not available in any of the PHC facilities in both rural and urban LGAs. Moduretic (amiloride & hydrochlorothiazide) tablet, the most recorded specific essential NCD medicines, was available in 5 (15.2%) of the PHC facilities in rural LGAs and none in the facilities in urban LGAs. The difference was not statistically significant. Essential medicines for CRDs such as salbutamol, beclomethasone and aminophylline drugs were found in not more than 2 (6.1%) of PHC facilities in the rural LGAs and none was available in the PHC facilities in the urban LGAs. Hydrocortisone injections and prednisolone tab were available in not more than 3 (9.1%) of PHC facilities in both rural and urban LGAs. Acetylsalicylic acid (vasoprin), a first line anti-platelet, was available in 2 (6.1%) of the PHC facilities in the rural LGAs and none in the urban LGAs. Statins, essential anti-lipid medicines were not recorded in any PHC facility.

**Table 4:** Comparison of availability of essential medicines for management of selected NCDs in PHC facilities in rural and urban LGAs

Essential medicines in PHC facilities	Rural *n* = 33		Urban *n* = 33		Statistics	
	Available	Not available	Available	Not available	Fisher’s exact test	*P*
**Essential medicines for hypertension**						
Methyldopa (Aldomet) tab	3 (9.1)	30 (90.9)	0 (0.0)	33 (100.0)	3.143	0.238
Lisinopril tab	1 (3.0)	32 (97.0)	0 (0.0)	33 (100.0)	1.015	1.000
Nifedipine tab	3 (9.1)	30 (90.9)	1 (3.0)	32 (97.0)	1.065	0.613
Propanolol tab	1 (3.0)	32 (97.0)	0 (0.0)	33 (100.0)	1.015	1.000
Hydroclorothiazide tab	1 (3.0)	32 (97.0)	0 (0.0)	33 (100.0)	1.015	1.000
Moduretic tab	5 (15.2)	28 (84.8)	0 (0.0)	33 (100.0)	5.410	0.053
**Essential medicines for diabetes**						
Any diabetes mellitus medicine^b^	0 (0.0)	33 (100.0)	0 (0.0)	33 (100.0)	-	-
**Essential medicines for chronic respiratory diseases (CRD)**						
Salbutamol (Ventolin) tab	2 (6.1)	31 (93.9)	0 (0.0)	33 (100.0)	2.063	0.492
Sabutamol aerosol inhaler	1 (3.0)	32 (97.0)	0 (0.0)	33 (100.0)	1.015	1.000
Beclomethasone aerosol inhaler	1 (3.0)	32 (97.0)	0 (0.0)	33 (100.0)	1.015	1.000
Aminophylline inj amp, IV	1 (3.0)	32 (97.0)	0 (0.0)	33 (100.0)	1.015	1.000
Prednisolone tab	3 (9.1)	30 (90.9)	2 (6.1)	31 (93.9)	0.216	1.000
Hydrocortisone inj. amp., IV	2 (6.1)	31 (93.9)	3 (9.1)	30 (90.9)	0.216	1.000
**Non-specific essential NCD medicines**						
Acetylsalicylic acid (Vasoprin) tab	2 (6.1)	31 (93.9)	0 (0.0)	33 (100.0)	2.063	0.492
Statins (Simva-or Atorvastatin) tab	0 (0.0)	33 (100.0)	0 (0.0)	33 (100.0)	-	-
Paracetamol tab	13 (39.4)	20 (60.6)	5 (15.2)	28 (84.8)	4.889^a^	0.027*

^a^Chi square test

^b^Any diabetes mellitus medicine: Metformin, glibenclamide (daonil) and insulin

^*^Statistically significant at *p* value < 0.05

-Statistics not generated because comparison group is zero

The preparedness of PHC facilities in the rural and urban LGAs to implement NCD essential interventions across the domains of staff training, basic equipment, diagnostics and essential medicines is shown in Table 5. The level of preparedness of PHC facilities in the rural and urban LGAs to implement essential NCD interventions across the domains was low. More than 90% (≥ 30) of the PHC facilities in both rural and urban LGAs were not prepared to implement essential interventions for each NCD across the component domains of staff training and essential medicines. The PHC facilities were better prepared across basic equipment domain than others. Eighty-eight percent of the PHC facilities in rural LGAs and 27 (81.8%) in the urban LGAs were prepared concerning availability of basic equipment for hypertension essential interventions. However, this is different for chronic respiratory disease where only 2 (6.1%) of PHC facilities in both rural and urban LGAs were prepared concerning availability of basic equipment respectively.

**Table 5:** Preparedness of PHC facilities in the rural and urban LGAs to implement essential NCD interventions

**Preparedness of PHC facilities across the domain of tracer items**	**Location**					
	**Rural** ***n*** ** = 33 (%)**		**Urban** ***n*** ** = 33 (%)**		**Fischer’s exact test**	***P***
**Domain**	**Prepared**	**Not prepared**	**Prepared**	**Not prepared**		
**Staff training**						
Hypertension	0 (0.0)	33 (100.0)	2 (6.1)	31 (93.9)	2.835	0.092
Diabetes	1 (3.0)	32 (97.0)	3 (9.1)	30 (90.9)	1.111	0.292
Chronic resp. diseases	0 (0.0)	33 (100.0)	0 (0.00)	33 (100.0)	-	-
**Basic equipment**						
Hypertension	29 (87.9)	4 (12.1)	27 (81.8)	6 (18.2)	0.474	0.491
Diabetes	26 (78.8)	7 (21.2)	29 (87.9)	4 (12.1)	0.992	0.319
Chronic resp. diseases	2 (6.1)	31 (93.9)	2 (6.1)	31 (93.9)	0.000	1.000
**Basic diagnostics**						
Diabetes	7 (21.2)	26 (78.8)	12 (36.4)	21 (63.6)	1.848^a^	0.174
**Essential medicines**						
Hypertension	2 (6.1)	31 (93.9)	0 (0.0)	33 (100.0)	2.835	0.092
Diabetes	0 (0.0)	33 (100.0)	0 (0.0)	33 (100.0)	-	-
Chronic resp. diseases	2 (6.1)	31 (93.9)	1 (3.0)	32 (97.0)	0.356	0.551
**Overall**						
Hypertension	2 (6.1)	31 (93.9)	1 (3.0)	32 (97.0)	0.356	0.551
Diabetes	0 (0.0)	33 (100.0)	3 (9.1)	30 (90.9)	4.302	0.038*
Chronic resp. diseases	0 (0.0)	33 (100.0)	0 (0.0)	33 (100.0)	-	-
**All 3 NCDs**	1 (3.0)	32 (97.0)	0 (0.0)	33 (100.0)	1.402	0.236

^a^Chi square test

*Statistically significant at *p* value < 0.05

-statistics not generated because comparison group is zero

NCDs- Non-communicable diseases

In terms of preparedness of the PHC facilities to implement essential interventions across all the domains, only 2 (6.1%) of the PHC facilities in rural LGAs and 1 (3%) in the urban LGAs were prepared for implementation of essential hypertension interventions; 3 (9.1%) of the PHC facilities in the urban LGAs and none in the rural LGAs was prepared for DM. There was no any PHC facility prepared for implementation of CRDs interventions in both rural and urban LGAs settings. Overall, across all component domains for the 3 selected NCDs, only 1 (3.0%) of the PHC facilities in the rural LGAs and none in urban LGAs was prepared for implementation of essential interventions for the 3 selected NCDs.

## Discussion

The availability of basic technologies for diagnosis and management of selected NCDs in PHC facilities in the rural and urban LGAs varied. In both rural and urban LGAs, manual blood pressure apparatus was available in about four-fifth of the PHC facilities. This was slightly higher than findings of a blood pressure measurement device in three-quarters of rural and urban primary level health facilities in Tanzania in 2014 [23]. In contrast to our study, a manual blood pressure apparatus is expected to be available in every PHC facility, similar to what was reported by Pakhare et al [30] in India and another study conducted across 8 LMICs by Mendis et al [19] where all the health facilities surveyed had at least one functional sphygmomanometer. Digital (automatic) blood pressure apparatus was available in one-third of PHC facilities in urban LGAs and twelve percent of PHC facilities in the rural LGAs. The availability of digital blood pressure apparatus was higher in this present study than the finding of one-tenth among selected health facilities across many LMICs where a study was conducted between 2009 and 2011 [19]. Higher penetration of basic technology over the years in LMICs including Nigeria may account for the availability of digital sphygmomanometer in higher proportion, especially in the urban LGAs in this present study.

The availability of basic diagnostic tests such as glucometer was slightly higher in PHC facilities in urban LGAs than the rural LGAs. The availability of various urine dipstick tests (glucose, protein and ketones), serum cholesterol and creatinine in PHC facilities was low in both the rural and urban LGAs. The low availability of basic diagnostic tests was supported by the findings from previous studies in many LMICs where major gaps in access to basic diagnostics had been reported [19, 30–32]. The basic technologies for essential interventions for chronic respiratory diseases were not available in PHC facilities in the present study. Studies carried out in LMICs have reported low availability of necessary equipment for control and management of chronic respiratory diseases and they focused on higher levels of care (secondary and tertiary) [33, 34].

The implication of these findings of inadequate availability of basic equipment and diagnostics for selected NCDs at the PHC facilities might include a hindrance to proper and timely screening services (individual and mass screening) for at risk populations at the PHC facilities and within their communities. Prompt diagnosis and commencement of early treatment where necessary for those diagnosed with selected NCDs will also be hampered. Also, due to inability to adequately recognize complicated NCD conditions at the PHC facilities, instituting timely referral services for those who will require it might also be affected.

Equitable access to essential medicines remains one of the potent strategies to address the increasing burden of chronic non-communicable diseases especially in the LMICs [35]. The present study showed that availability of essential medicines for implementation of essential NCD interventions was very low in PHC facilities in the rural and urban LGAs. This was in contrast to other studies by Mendis et al [36] in Nigeria, Peck et al [23] in Tanzania, Pakhare et al [30] in India and Mendis et al [19] in a survey conducted in eight low- and middle-income countries where anti-hypertensive drugs were found in about half or more of the primary level health facilities surveyed. Minh et al [37], reported that thiazide diuretic was among most commonly available essential NCD medicines in primary level health facilities in Vietnam, similar to the finding in this present study where moduretic was the most available essential NCD medicine.

The essential medicines for diabetes were not available in any of the PHC facilities in both rural and urban LGAs. This was supported by the report of non-availability of anti-diabetics- insulin in any PHC facility in 4 of the 8 LIMCs where survey was conducted [19]. In contrast, many studies in literature have reported availability of oral anti-diabetic drugs to varying degree [19, 23]. Pakhare et al [30] in India reported that first line drug (metformin) for management of diabetes mellitus was available in more than two-thirds of all the PHC facilities surveyed. Other drugs used in management of diabetes mellitus (other oral drugs and insulin) were less available than metformin [30].

Essential medicines used in management of chronic respiratory diseases such as salbutamol, beclomethasone and aminophylline were found in very few PHC facilities in the rural LGAs and none in the urban LGAs. This was in contrast with the findings from 2012 Tanzania Service Availability and Readiness Assessment (SARA) where salbutamol and beclomethasone inhalers were recorded in about one-quarter and fourteen percent of health facilities in the rural areas respectively and about half and fifteen percent in the urban areas respectively [31]. Hydrocortisone injections and prednisolone tablets were recorded in less than one tenth of PHC facilities in both rural and urban LGAs. This was slightly lower than the findings of about one-quarter and two-third of the health facilities in the rural and urban areas respectively in the Tanzania survey [31].

The low availability of essential medicines for implementation of essential NCD interventions in the PHC facilities in rural and urban LGAs of Osun state might be as a result of poor government support for the PHC system. The findings of low availability of essential NCD medicines in PHC facilities and giving prescriptions to patients to buy at unregulated drug stores pose risk to the health of people with selected NCDs as it might cause poor adherence to medication and care, lack of confidence in the health system and lead to seeking alternative care with attendant untoward consequences.

In this present study, the level of preparedness of PHC facilities in the rural and urban LGAs to implement essential NCD interventions across the four component domains of service preparedness was very low. This was similar to findings in several studies in many low resource countries which reported that the PHC systems were not adequately prepared to cope with the rising burden of major NCDs in their countries [23, 30, 38–41]. A study that used secondary data from the Tanzanian national service provision survey reported that only 28% of health centres and dispensaries were prepared for the outpatient primary care of hypertension [39]. The findings from the Tanzanian study had a higher level of preparedness than what was found in this present study because it focused on hypertension only.

In our study, using 50% availability of the tracer essential medicines, basic technologies and staff training for the three NCDs, only 1 (3.0%) of the PHC facilities in the rural LGAs and none in urban LGAs were prepared on implementation of essential NCD interventions. The PHC facilities in rural and urban LGAs in this present study were far from the target of WHO Global Action Plan, an 80% availability of the affordable basic technologies and essential medicines (including generics) required to treat major non-communicable diseases in both public and private facilities [15]. This target is to be achieved by 2025 [15]. For future studies, the authors recommend 80% availability cut-off, in line with the relevant targets of WHO Global NCD Plan.

The PHC facilities in both rural and urban LGAs were least prepared in terms of availability of essential medicines followed by the staff training domain and then basic diagnostics domain. The PHC facilities in both LGA settings were only better prepared in terms of availability of basic equipment for hypertension and diabetes mellitus but not prepared for chronic respiratory diseases. A similar finding was reported by Pakhare et al [30] where the top three domains (essential medicines, staff training and basic diagnostics) reported in our study were found to be major domains where primary care facilities were less prepared for hypertension and other cardiovascular disease services [30]. The study reported availability of necessary items was least in diagnostic services, human resource domain and followed by essential drugs, and reported better in domains of equipment and point-of-care supply [30].

The very low level of preparedness of PHC facilities in the rural and urban LGAs to implement essential NCD interventions reported in our study may limit the capacity of PHC facilities to contribute significantly to the control and management of selected NCDs among people in communities where they live and work. This will hamper health promotion and preventive activities such as screening for NCDs among at risk populations, and using essential NCD medicines to control non-complicated hypertension, DM and chronic respiratory diseases such as asthma.

This present study made an important contribution to the assessment of health facility survey on preparedness for essential NCD interventions. While few previous studies determined preparedness [23, 39] or capacity [19] of primary level health facilities to offer essential services for selected NCDs on individual basis and only across each component domain, this study, in addition, established an overall level of preparedness across all the domains combined for the three selected NCDs. This is a major strength of our study.

### Limitation of the study

This study has few limitations. The self-reported nature of the responses might introduce bias. This was minimized by assuring the respondents of absolute confidentiality. The direct observation of the items in the facilities also helped to reduce bias on the availability of basic equipment, diagnostics, and essential medicines.

## Conclusion

The availability of basic diagnostics and essential medicines in PHC facilities in the rural and urban LGAs for essential NCD interventions is low. The basic equipment for diagnosis and management of hypertension were available in most PHC facilities, but those for chronic respiratory diseases were not available in the PHC facilities in both rural and urban LGAs settings. Availability of basic diagnostic tests in PHC facilities was slightly higher in urban LGAs than the rural LGAs. Essential medicines for treatment and control of selected NCDs were barely available in the PHC facilities in both rural and urban LGAs. Only few PHC facilities had antihypertensives and drugs for CRDs, while none had essential drugs for DM. Majority of the PHC facilities in rural and urban LGAs were not prepared for implementation of essential NCD interventions. The PHC facilities in both rural and urban settings were least prepared in terms of availability of essential medicines followed by the staff training and then basic diagnostics.

### Recommendations

We thus recommend that the state government should strengthen the PHC system by providing needed essential medicines, basic diagnostics, equipment and training of clinical health care workers for implementation of essential NCD interventions in the state. The SPHCDB and heads of PHC facilities should set up mechanisms such as Drug and Supply Revolving Fund, etc. to prevent stock out of basic diagnostic dipsticks, glucometer strips, and essential medicines. This will help to ensure continuous delivery of effective essential NCD interventions to the people. For further study, this study should be replicated to determine level of preparedness of PHC facilities on implementation of essential NCD interventions across states in various regions of Nigeria. The study design and methodology could also be adapted to assess level of preparedness of other health care levels (secondary and tertiary), public and private health facilities on implementation of essential NCDs intervention in Osun State and other parts of the country. This will guide health system strengthening to improve response to the increasing burden of NCDs in the state and the country at large.

## Supplementary Information

**Additional file 1: **Appendix I. Tracer Items and Domains for PHC Facility NCD Service Readiness Assessment. Appendix 2. Comparison of availability of trained staff, IEC materials and guidelines in rural and urban PHCfacilities for implementation of essential NCD interventions.

## Data Availability

Datasets supporting the findings of this study are available from the corresponding author, A.F.A, on request.

## Abbreviations

BP: Blood pressure
CRD: Chronic respiratory diseases
CVD: Cardiovascular disease
DM: Diabetes mellitus
IECs: Information education and communication
LGA: Local Government Area
LMICs: Low- and middle-income countries
MOH: Medical Officer of Health
NCD: Non-communicable Disease
PHC: Primary Health Care
SARA: WHO Service Availability and Readiness Assessment
WHO: World Health Organization
WHO PEN: WHO Package of Essential Non-communicable Disease Interventions
