# Supplementary material for: Preparedness of primary health care facilities on implementation of essential non-communicable disease interventions in osun state south-west Nigeria: a rural–urban comparative study
Source: BMC Health Serv Res. 2023 Feb 14;23:154. doi: 10.1186/s12913-023-09138-8 (PMC9930353; doi:10.1186/s12913-023-09138-8)
Supplement: Supplementary file 1 — Additional file 1: Appendix I. Tracer Items and Domains for PHC Facility NCD Service Readiness Assessment. Appendix 2. Comparison of availability of trained staff, IEC materials and guidelines in rural and urban PHCfacilities for implementation of essential NCD interventions. [file 12913_2023_9138_MOESM1_ESM.docx]

**Appendix I**

**Tracer Items and Domains for PHC Facility NCD Service Readiness Assessment**

| s/n | **Domain** | **Diabetes** | | **Hypertension (Cardiovascular disease)** | | **Chronic respiratory disease** | | **Overall facility readiness** |
| --- | --- | --- | --- | --- | --- | --- | --- | --- |
| 1 | **Staff & Training** | Trained staff |  | Trained staff |  | Trained staff |  |  |
|  |  | IEC |  | IEC |  | IEC |  |  |
|  |  | Guidelines |  | Guidelines |  | Guidelines |  |  |
| 2 | **Basic equipment** | Adult scale |  | Adult scale |  | Peak flow metre |  |  |
|  |  | Tape (Stadiometre/ Height board) |  | Stethoscope |  | Stethoscope |  |  |
|  |  | Blood Pressure apparatus |  | Blood Pressure apparatus |  | Spacer for inhaler or nebulizer |  |  |
| 3 | **Basic Diagnostics** | Blood Glucose |  |  | | | |  |
|  |  | Urine dipstick protein |  |  |  |  |  |  |
|  |  | Urine dipstick ketones |  |  |  |  |  |  |
| 4 | **Essential medicines** | Metformin |  | ACE inhibitors |  | Salbutamol |  |  |
|  |  | 2nd gen Sulphonylureas |  | Thiazides |  | Beclomethasone |  |  |
|  |  | Daily insulin |  | Beta blockers |  | Predinisolone |  |  |
|  |  | Glucose solution |  | Calcium channel blockers |  | Hydrocortisone |  |  |
|  |  |  |  | Aspirin |  |  |  |  |
|  | **Readiness summary** | % across all domains | | % across all domains | | % across all domains | | % across all the diseases |

**A score of 1 was assigned to each item in the component domains and the mean score express as percentage*

IEC- Information, education and communication materials displayed; NCD- Non-communicable disease

**Appendix 2:**

**Comparison of availability of trained staff, IEC materials and guidelines in rural and urban PHC facilities for implementation of essential NCD interventions**

| **Availability of a trained staff, IEC material and guideline in PHC facilities** | **Location** | | | **Statistics**  **χ2** |
| --- | --- | --- | --- | --- |
|  | **Rural**  **n = 33 (%)** | **Urban**  **n =33 (%)** |  |  |
| **Trained Staff**  **Hypertension**  Staff training in the last 2 years  No recent training | 3 (9.1)  30 (90.9) | 6 (18.2)  27 (81.2) |  | 1.158**^+^**  p= 0.475 |
| **Diabetes mellitus**  Staff training in the last 2 years  No recent training | 5 (15.2)  28 (84.8) | 9 (27.3)  24 (72.7) |  | 1.451  p= 0.228 |
| **Chronic respiratory diseases**  Staff training in the last 2 years  No recent training | 1 (3.0)  32 (97.0) | 2 (6.1)  31 (93.9) |  | 0.349**^+^**  p= 1.000 |
| **Guideline**  **Hypertension**  Available  Not available | 4 (12.1)  29 (87.9) | 4 (12.1)  29 (87.9) |  | 0.000  p= 1.000 |
| **Diabetes mellitus**  Available  Not available | 1 (3.0)  32 (97.0) | 2 (6.1)  31 (93.9) |  | 0.349**^+^**  p= 1.000 |
| **Chronic respiratory diseases**  Available  Not available | 3 (9.1)  30 (90.9) | (0 (0.0)  33 (100.0) |  | 3.143**^+^**  p= 0.238 |
| **IEC Material**  **Hypertension**  Available  Not available | 0 (0.0)  33 (100.0) | 3 (9.1)  154 (77.0) |  | 3.143**^+^**  p= 0.238 |
| **Diabetes mellitus**  Available  Not available | (0 (0.0)  33 (100.0) | 7 (21.2)  26 (78.8) |  | 7.831**^+^**  p= 0.011* |
| **Chronic respiratory diseases**  Available  Not available | (0 (0.0)  33 (100.0) | 1 (3.0)  32 (97.0) |  | 1.015**^+^**  p= 1.000 |
| **Shared risk factors#**  Available  Not available | 6 (18.8)  27 (81.8) | 10 (30.3)  23 (69.7) |  | 1.320  P= 0.251 |

**+** Fisher’s exact test; * statistically significant at p value < 0.05

# Shared risk factors include tobacco use, unhealthy diet, physical inactivity and harmful use of alcohol.

The comparison of tracer items for staff training domain (trained staff, IEC materials and guidelines) in rural and urban PHC facilities for implementation of essential NCD interventions is shown in Table 7. The PHC facilities in urban and rural LGAs had more staff attended training in last 2 years on diabetes mellitus (DM) and IEC materials on DM. Twenty seven percent of the PHC facilities in urban LGAs had staff trained on DM in last two years more than 5 (15.2%) of the PHC facilities in the rural LGAs. The difference was not statistically significant, p= 0.228. The information, education and communication (IEC) materials for diabetes mellitus were available in 7 (21.2%) of PHC facilities in urban LGAs and none was available in the rural areas. This difference was statistically significant, Fisher’s exact test = 7.831, p = 0.011. The guidelines for management of the 3 NCDs were not available in majority of the PHC facilities in both rural and urban LGAs settings.
